# Inguinal tumorous mass – an uncommon late manifestation of chronic organized hematoma after laparoscopic transabdominal preperitoneal inguinal hernia repair: Two cases report

**DOI:** 10.1016/j.ijscr.2019.12.016

**Published:** 2019-12-17

**Authors:** Petr Chmatal, Radan Keil

**Affiliations:** Institute of Aviation Medicine Prague, Generala Piky 1, 160 00 Praha 6, Czech Republic

**Keywords:** TAPP, transabdominal preperitoneal inguinal hernia repair, COH, chronic organized hematoma, ASA, American Society of Anesthesiologist, CEH, chronic expanding hematoma, Transabdominal preperitoneal inguinal hernia repair, Chronic hematoma, Tumour, TAPP, Rare disease

## Abstract

**Background:**

Hematoma is a common complication following inguinal hernia repair. It is usually diagnosed early after surgical procedure and is spontaneously, or after evacuation, resorbed. Chronic organized hematoma is rare surgery complication, there is no relevant article after laparoscopic hernia repair in literature.

**Cases presentation:**

62- and 54-year-old men presented a groin palpable tumorous mass several month after uncomplicated laparoscopic transabdominal preperitoneal inguinal hernia repair. Their dominant complaints were mechanical restrictions. The ultrasound showed a solid formation subcutaneously. The removal and histopathology examination confirmed a chronic organized hematoma and at the same time, this ended the patients ‘problems.

**Conclusion:**

Both patients represent uncommon cases of late postoperative difficulties, uncharacteristic localization of chronic organized hematoma and the need for surgical reintervention to exclude neoplasm.

## Highlights

• Chronic organized hematoma is rare surgery complication, it is uncommon after laparoscopic hernia repair especially.
• Two patients were re-operated on with groin tumorous mass in longer intervals after transabdominal preperitoneal inguinal hernia repair from the anterior access.
• Tumorous bulk outside of postoperative scars and outside original operating space was made up of old organized hematoma.
• Only removal of tumour could exclude neoplasm and ends patient’s mechanical restrictions.

## 1 Background

Inguinal hernia repair is one of the most frequent surgical procedures in general surgery around the world. For male patients with primary unilateral inguinal hernia a laparoscopic technique is suggested [1]. Postoperative complications are watched and evaluated. The incidence of hematoma after laparoscopic transabdominal preperitoneal inguinal hernia repair (TAPP) is reported from 3 to 8%. The latest published meta-analysis puts the incidence at 3,4% on average [2]. The risk of hematoma is increased by partially absorbable mesh, chronic anticoagulation, recurrent hernia procedure, mesh fixation, larger hernia defect and medial defect localization [[3], [4], [5]]. Hematomas commonly manifest themselves early after the surgical procedure and imaging examinations show a fluid collection. Although hematoma as a fluid collection after TAPP is not unusual, the finding of chronic organized hematoma (COH) as a hard inguinal mass causes mechanical problems and solid formation image on ultrasound is rare. This work has been reported in line with the SCARE criteria [6].

## 2 Cases presentation

### 2.1 Case report

**Fig. 1:**
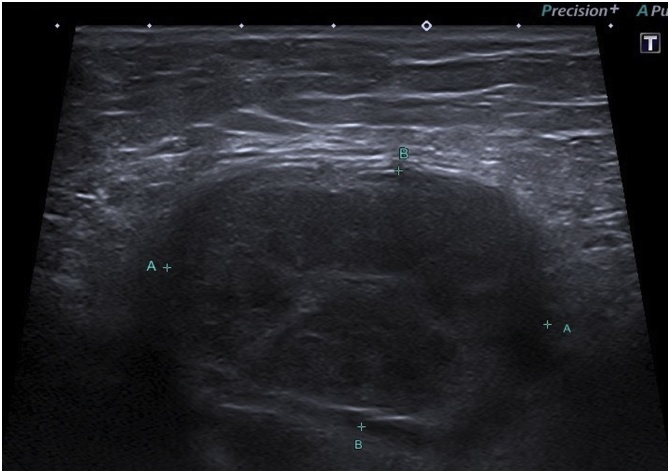
Chronic organized hematoma – ultrasound image eight month after TAPP.

**Fig. 2:**
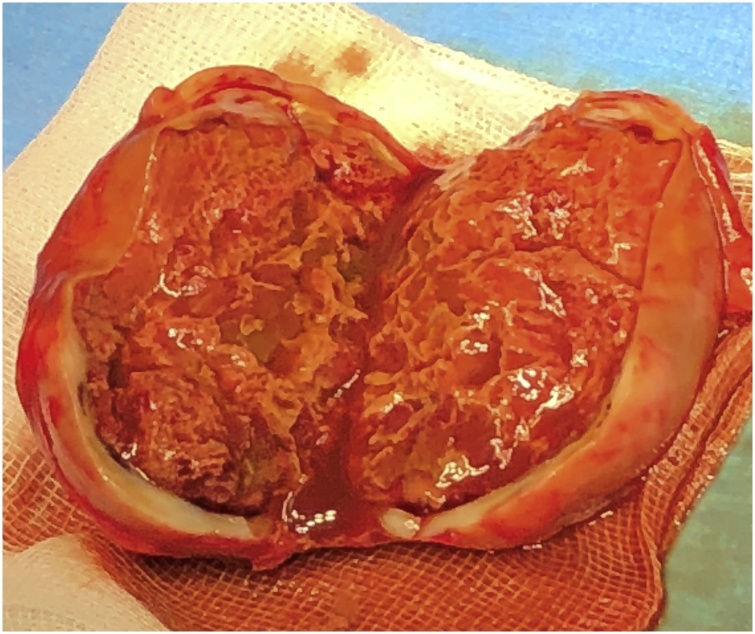
Chronic organized hematoma – macroscopic image.

**Fig. 3:**
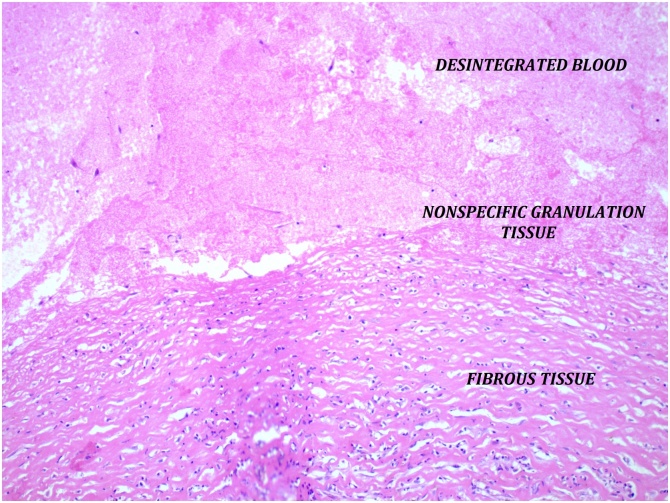
Chronic organized hematoma – microscopic image.

A 62-year old white man had a left inguinal hernia, 5 × 2 cm in size, which lasted for two years. He was suffering from pain and pressure in the groin. With regard to additional diseases, there was only hypertension treated with angiotensin blockers. Clinical finding apart from hernia was physiological, ASA classification II. TAPP procedure lasted 60 min without any complications. We identified direct hernia. The inguinal defect was covered using polypropylen non-absorbable mesh which was fixed with ProTack (5 mm) on Cooper’s ligament and transverse abdominal muscle. The patient was discharged to home care the next morning. The stitches were removed after 8 days. The last follow-up was after 4 weeks without any complaints and with normal postoperative status. The patient returned to surgery four month later with a palpable tumour in the groin, 4 × 6 cm in diameter. The ultrasound picture repeatedly showed an uncharacteristic formation, presumably of solid hematoma or tumorous lymphatic node subcutaneously (Fig. 1). The puncture was unsuccessful. The tumour was removed eight months after the TAPP from anterior access (Fig. 2). The inguinal channel was firm and intact. The patient was discharged same day and postoperative course was without any complications. Macroscopic and histopathology findings confirmed COH (Fig. 3). The patient is constantly without problems.

### 2.2 Case report

A 54-year-old white man had a left inguinal hernia, lasting for fifteen years, 6 × 6 cm in size. The hernia limited him in hard work. He had no additional diseases in history. Clinical finding apart from hernia was physiological, ASA classification I. The TAPP procedure lasted 60 min without any complications. We identified the indirect hernia sac. The non-absorbable mesh was used into preperitoneal space which was fixed with ProTack (5 mm) on Cooper’s ligament and transverse abdominal muscle. The patient was discharged to home care the next morning. The stitches were removed after 7 days. The last follow-up after 3 weeks was without any complaints and with a corresponding postoperative finding. This patient returned to surgery after 10 weeks with subcutaneous firm bulk limiting his motion. The ultrasound finding was suspicious of COH. The puncture was unsuccessful. The surgery was done three months after the TAPP from the anterior access, the inguinal channel was firm and we removed a tumour, 3 × 2 cm in size. The patient was discharged same day and postoperative course was without any complications. Macroscopic and histopathology findings confirmed COH. The patient is constantly without problems.

## 3 Discussion and conclusions

The chronic organized hematoma is a rare complication and can occur in a variety of postoperative or post-traumatic locations. It is a tough and fibrous tissue mass with an uncharacteristic view on imaging examinations. The diagnosis is often difficult and it can be easily mistaken for one of the relatively more frequent malignant neoplasms. The clear mechanism responsible for the development of COH remains unclear. After initial bleeding, chronic changes of hematoma typically start – fibroblasts create inner and outer membranes during the process of local inflammation – hematoma encapsulate with a central mass of blood, a wall of granulation tissue and fibrous tissue at the periphery. In case of COH fibrous septs grow inside the capsule during the following several weeks and contain highly fragile neocapillary vessels. Repeated bleeding from these neocapillars causes irritant effects of blood and self-expansion of hematoma supports fibroblast activities. It results in the genesis of fibrous trabecular structure with small lacunas of liquid hematoma inside the membranes or fibrous material slowly increase in volume, forming a solid hematoma, in which the inner and outer membranes tend to fuse completely. This process can continue to deposit calcium to tissue. Macroscopic and microscopic findings of COH are associated with reactive inflammation and it is analogous with the chronic expanding hematoma (CEH) described by Reid in 1980 [7]. There is no consensus on the diagnostic term of this disease’s entity.

Both our described cases did not presage any complications. The patients came for the follow-up in long interval after their uncomplicated TAPP with mechanical problems caused by inguinal formations, which were outside of postoperative scars and outside direct operating space. Though patients were operated on by different surgeons, the TAPP technique is standardized in our facility and should not influence the healing process. CT or MRI examination before reoperations could not bring any additional information and wasn’t used. Although exists studies which recommend laparoscopy in inguinal hernia reoperations [8], in our cases a laparoscopic approach couldn’t get to tumour above abdominal wall.

The COHs in our patients after TAPP occurred out of direct surgery area, caused mechanical restriction, had uncharacteristic finding on imaging examinations and remove was needed to exclude neoplasm. Inguinal channel histopathology changes in men with inguinal hernia was precisely described in studies from University of Palermo [[9], [10], [11]]. Postoperative changes are described in several studies. We didn’t search any relevant articles concerning COH or CEH after TAPP in the literature.

## Sources of funding

No funds were provided.

## Ethical approval

The ethical approval for this case report is exempt from ethical approval in our institution.

## Consent

Written consent for publication of their clinical details and images was obtained.

## Author contribution

Petr Chmatal: Recources, Writing - Original Draft. Radan Keil: Writing - Review and Editing.

## Registration of research studies

Not applicable.

## Guarantor

Corresponding author.

## Provenance and peer review

Not commissioned, externally peer-reviewed.

## Declaration of Competing Interest

The authors declare that they have no competing interests.
